# TMEM160 promotes hepatocellular carcinoma cell proliferation, invasion, and immune evasion by regulating the VEGFA/PI3K/AKT signaling axis

**DOI:** 10.3389/fimmu.2026.1829095

**Published:** 2026-06-16

**Authors:** Guodong Shi, Dehua Liu, Jinhan Qiao

**Affiliations:** 1 Department of Interventional Therapy, Cancer Hospital of Dalian University of Technology, Shenyang, China; 2 Department of Interventional Therapy, Liaoning Cancer Hospital, Shenyang, China; 3 Department of Interventional Therapy, Liaoning Cancer Institute, Shenyang, China

**Keywords:** angiogenesis, hepatocellular carcinoma, immune evasion, PI3K/AKT, TMEM160, VEGFA

## Abstract

**Objective:**

Hepatocellular carcinoma (HCC) remains a major cause of cancer-related mortality, and TMEM160 is potentially involved in tumor progression and immune escape. This study aimed to determine the expression pattern and clinical relevance of TMEM160 in HCC.

**Methods:**

TMEM160 expression was analyzed in public HCC cohorts and validated in 76 paired HCC tissues and adjacent non-tumor tissues. Survival associations were assessed using Kaplan–Meier analysis. TMEM160 was silenced in Huh7 and Hep3B cells and overexpressed in SNU449 cells, followed by assays of proliferation, invasion, and endothelial tube formation using HUVECs conditioned medium. PD-L1 protein expression was detected by Western blotting. HCC cells were co-cultured with PBMCs, followed by ELISA detection of IFN-γ, IL-2, IL-10, and TGF-β secretion and flow cytometric analysis of CD8^+^IFN-γ^+^ T cells to evaluate immune-related effects. Mechanistically, PI3K and AKT phosphorylation changes were examined, and VEGFA rescue experiments were performed by overexpressing VEGFA in TMEM160-silenced HCC cells. Additionally, a subcutaneous murine tumor model was used to validate *in vivo* effects, and tumor tissues were examined by H&E staining, immunohistochemistry, and Western blotting.

**Results:**

TMEM160 was increased in HCC tissues and HCC cells, and high TMEM160 expression was associated with poorer overall survival. TMEM160 knockdown reduced HCC cell viability, invasion, HUVEC tube formation with decreased VEGFA expression. TMEM160 knockdown also decreased PD-L1 protein levels in HCC cells. TMEM160 modulation was accompanied by concordant changes in PI3K/AKT phosphorylation, and VEGFA overexpression increased PI3K/AKT phosphorylation and partially rescued inhibitory phenotypes caused by TMEM160 silencing. *In vivo*, TMEM160 knockdown suppressed tumor growth, reduced TMEM160-positive and VEGFA-positive proportions, increased interferon-γ (IFN-γ) positivity, and decreased p-PI3K/PI3K and p-AKT/AKT ratios in tumor tissues.

**Conclusion:**

These findings supported that TMEM160 might affect VEGFA-associated PI3K/AKT signaling to promote malignant phenotypes in HCC, suggesting TMEM160 as a candidate molecular target for further investigation.

## Introduction

Primary liver cancer continues to cause considerable morbidity and mortality worldwide (1, 2). Despite advances in surveillance and multidisciplinary management, long-term outcomes are still constrained by the intrinsically aggressive biology of hepatocellular carcinoma (HCC), with post-curative recurrence remaining common. Clinical practice guidelines report that recurrence can approach 70% within 5 years after resection or ablation in selected patients (3, 4). In parallel, systemic therapy has rapidly evolved into an era characterized by molecularly targeted agents and immune-based regimens, including first-line immunotherapy combinations supported by pivotal phase III trials (5, 6). Nevertheless, objective responses and durable benefit are achieved only in a subset of patients, and marked interpatient variability in treatment sensitivity and disease course persists, underscoring the need to identify additional key molecular drivers that can better explain malignant progression and immune escape (7).

TMEM160 is a member of the transmembrane protein (TMEM) family. TMEM proteins are located in different biological membranes and contain at least one transmembrane region. Although many TMEM proteins remain incompletely characterized, available evidence indicates that they participate in diverse physiological processes required for cellular homeostasis, such as glycosylation, cardiomyocyte development, epidermal keratinization, and autophagy. In cancer, TMEM family members have attracted increasing attention because transcriptomic and proteomic studies have revealed differential expression of various TMEM proteins between tumor and non-tumor tissues (8). Functionally, TMEM proteins have been implicated in the promotion or suppression of tumor cell proliferation, epithelial–mesenchymal transition, migration, invasion, metastasis, modulation of immune responses, and responses to antineoplastic drugs (8). Available basic evidence suggests that TMEM160 is linked to mitochondrial stress homeostasis, as its depletion disrupts redox balance and activates the mitochondrial unfolded protein response, and it has also been implicated in protein networks related to mitochondrial quality control (9, 10). Although the overall literature is still sparse, emerging studies in other malignancies have begun to suggest pro-tumorigenic roles for TMEM160. In colorectal cancer, TMEM160 has been reported to interact with and stabilize PD-L1, thereby facilitating immune evasion and radioresistance (11). In lung adenocarcinoma and cervical adenocarcinoma models, TMEM160 knockdown suppresses tumor cell proliferation and provides clues to its potential interaction network (12). However, to our knowledge, evidence regarding TMEM160 in HCC is still scarce, and its functional role and potential downstream mechanisms in HCC warrant further investigation.

HCC progression is driven by coordinated malignant phenotypes, including sustained tumor cell proliferation, enhanced migratory and invasive capacity, and robust tumor angiogenesis that supports rapid growth and dissemination (13, 14). Among pro-angiogenic signals, VEGFA is widely recognized as a central mediator of neovascularization and has repeatedly been positioned as a key actionable node in HCC (15). Consistent with this concept, studies have reported that upstream regulators can promote angiogenesis through the RhoB–VEGFA–VEGFR2 axis in association with adverse outcomes, and that post-transcriptional mechanisms such as increased VEGFA mRNA stability can further augment pro-angiogenic activity in HCC models (16, 17). In parallel, the PI3K/AKT pathway represents a prototypical pro-survival and pro-metastatic signaling cascade frequently implicated in HCC, supporting tumor cell growth and survival and contributing to EMT-related changes that facilitate aggressive behavior (18, 19). Notably, some experimental studies have observed that suppression of invasion and endothelial tube formation occurs alongside downregulation of VEGF/VEGFR2 signaling and inhibition of PI3K/AKT activity, suggesting a functional interplay between these axes in shaping angiogenesis- and invasion-related phenotypes (20). Therefore, delineating how upstream determinants modulate VEGFA/PI3K/AKT-associated processes may provide a clearer mechanistic basis for HCC malignancy and help identify potential intervention points.

Accordingly, this study aimed to determine the expression pattern and clinical relevance of TMEM160 in HCC and to define its functional roles in HCC malignant phenotypes, including proliferation, invasion, angiogenesis, and immune evasion. In addition, potential downstream mechanisms associated with TMEM160-mediated HCC progression were further explored.

## Methods

### Bioinformatics analysis

Publicly available transcriptomic datasets were used to analyze TMEM160 expression in HCC. RNA-seq expression data and corresponding clinical information for TCGA_LIHC were obtained from The Cancer Genome Atlas through the GDC Data Portal. GEO datasets GSE14520 and GSE54236 were obtained from the NCBI Gene Expression Omnibus database. TMEM160 expression was compared between HCC tissues and normal or adjacent non-tumor liver tissues in these cohorts. For pathway enrichment analysis, gene set enrichment analysis (GSEA) was performed using the BEST application (Biomarker Exploration for Solid Tumors) to explore signaling pathways associated with TMEM160 expression in HCC.

### Patients and clinical tissue specimen collection

This study consecutively enrolled 76 patients with histologically confirmed HCC who underwent curative-intent surgical resection at our hospital between 2019 and 2020. None of the enrolled patients received chemotherapy, radiotherapy, targeted therapy, or immunotherapy prior to tissue collection. Patients with a history of other malignancies, recurrent HCC, or incomplete clinicopathological information were excluded. For each patient, tumor tissues and matched adjacent non-tumor liver tissues were collected intraoperatively at the time of surgical resection. Adjacent non-tumor tissues were obtained from areas distant from the tumor margin and were confirmed to be free of tumor involvement by pathological examination.

Age, sex, lymph node involvement, tumor size, TNM stage, and hepatitis B virus (HBV) infection status were collected from medical records according to standardized definitions. All patients were followed for 5 years after surgery through outpatient visits and telephone interviews. Survival status and follow-up time were recorded. This study was conducted in accordance with the Declaration of Helsinki and was approved by the Ethics Committee of our hospital.

### Cell lines and cell culture

Human HCC cell lines (Huh7, Hep3B, HCCLM3, and SNU449), THLE-2 cells, and HUVECs were obtained from ATCC (Manassas, VA, USA). HCC cells were cultured in DMEM with 10% FBS, THLE-2 cells in RPMI-1640 with 10% FBS, and HUVECs in ECM with 10% FBS. All cells were maintained at 37 °C in 5% CO_2_ and collected during the logarithmic growth phase for subsequent experiments.

### PBMC isolation and co-culture

PBMCs were isolated from peripheral blood of healthy adult donors after informed consent and ethics approval using Ficoll-Paque density-gradient centrifugation. Briefly, anticoagulated blood was diluted with PBS (1:1), layered over Ficoll, and centrifuged at 400g for 30min at room temperature without brake. The interphase PBMC layer was collected and washed twice with PBS (300g for 10min). PBMCs were resuspended in RPMI-1640 supplemented with 10% FBS, and preparations with viability >90% (trypan blue exclusion) were used. For co-culture, HCC cells from the predefined experimental groups were seeded and allowed to adhere overnight. PBMCs were added at an effector-to-target ratio of 10:1 and co-cultured for 48h at 37 °C with 5% CO_2_. Supernatants were collected, clarified by centrifugation, and stored at −80 °C until analysis.

### Cell transfection

For cell transfection, lentiviral short hairpin RNA constructs targeting TMEM160 (sh-TMEM160#1, sh-TMEM160#2, and sh-TMEM160#3) and the corresponding negative control (sh-NC), as well as lentiviral vectors for TMEM160 overexpression (LV5-TMEM160) and the empty vector control (LV5-NC), together with the VEGFA overexpression plasmid (pcDNA3.1-VEGFA) and its blank vector (pcDNA3.1), were prepared by GeneChem Corp., Shanghai, China.

Cells were transfected with 5nM shRNAs, plasmids, or corresponding negative controls using Lipofectamine 3000 (Invitrogen, USA) in serum-free Opti-MEM according to the manufacturer’s protocol.

### CCK-8

Cell viability was evaluated with CCK-8 (Dojindo, Japan) following the manufacturer’s instructions. HCC cells (5×10³/well) were seeded in 96-well plates and incubated overnight. After treatment, 10 μL of CCK-8 solution was added to each well and incubated at 37 °C for 2 h. Absorbance at 450 nm was recorded using a microplate reader (BioTek, USA).

### Transwell assay

Cell invasion was assessed using Matrigel-coated Transwell chambers (8.0µm). Cells were seeded into the upper chamber in serum-free medium, and medium containing 10% FBS was added to the lower chamber. After 24h of incubation, non-invading cells were removed, whereas invaded cells were fixed, stained with crystal violet, photographed, and counted in five random fields under an inverted microscope.

### Tube formation assay

HUVECs were incubated with the corresponding conditioned medium before tube formation. Briefly, Matrigel was thawed on ice, added to pre-chilled 96-well plates (50µL/well), and allowed to polymerize at 37 °C for 30min. HUVECs were harvested and resuspended in the corresponding culture medium, then seeded onto the Matrigel-coated wells at a density of 2×10^4^ cells/well. After incubation at 37 °C with 5% CO_2_ for 6h, tube-like structures were observed and photographed under an inverted microscope. Quantification was performed by measuring the total tube length and number of branch points from five randomly selected fields per well using ImageJ.

### ELISA

IFN-γ, IL-2, IL-10, and TGF-β levels in culture supernatants were measured using ELISA kits (MyBioSource, USA) according to the manufacturer’s instructions. After co-culture, supernatants were collected, incubated in ELISA plates with the corresponding reagents, and read at 450nm. Cytokine concentrations were determined from standard curves.

### Flow cytometry

The proportion of CD8^+^IFN-γ^+^ cells in PBMCs after co-culture was analyzed by flow cytometry. Briefly, PBMCs were collected after 48 h of co-culture and washed twice with cold PBS. Cells were stained first with FITC-conjugated anti-human CD8a antibody (clone RPA-T8; BioLegend, Cat. No. 301006) at 4 °C in the dark. After surface staining, cells were fixed and permeabilized using the BD Cytofix/Cytoperm™ Fixation/Permeabilization Kit (BD Biosciences, Cat. No. 554714) according to the manufacturer’s instructions, followed by intracellular staining with PE-conjugated anti-human IFN-γ antibody (clone 4S.B3; BioLegend, Cat. No. 502509). Data were acquired using a BD FACSCanto™ II flow cytometer (BD Biosciences, San Jose, CA, USA) and analyzed with FlowJo software version 10.8.1 (BD Biosciences). CD8^+^IFN-γ^+^ cells were identified by sequential gating on lymphocytes based on forward scatter and side scatter, followed by gating on CD8^+^ cells and then IFN-γ^+^ cells. The percentage of CD8^+^IFN-γ^+^ cells was determined by sequential gating on lymphocytes and CD8^+^ cells.

### Co-immunoprecipitation assay

Co-immunoprecipitation was performed to examine the association between TMEM160 and VEGFA in Huh7 cells. Briefly, Huh7 cells were lysed with ice-cold IP lysis buffer containing protease inhibitor cocktail. After centrifugation at 12, 000 g for 15 min at 4 °C, the supernatants were collected and incubated with anti-TMEM160 antibody (Proteintech, 26451-1-AP), anti-VEGFA antibody (Abcam, ab214424), or normal rabbit IgG at 4 °C overnight. Subsequently, Protein A/G agarose beads were added to the lysates and incubated for another 2 h at 4 °C. The beads were washed three times with IP lysis buffer, and the immunoprecipitated protein complexes were eluted by boiling in SDS loading buffer. The input lysates and immunoprecipitated proteins were then analyzed by Western blotting using anti-TMEM160 and anti-VEGFA antibodies.

### Cycloheximide chase assay

To evaluate VEGFA protein stability, Huh7 cells transfected with sh-NC or sh-TMEM160 were treated with cycloheximide (CHX; 40 μg/mL; MedChemExpress, HY-12320) for 0, 2, 4, and 8 h. Total proteins were extracted at each indicated time point and subjected to Western blotting for VEGFA, TMEM160, and β-actin. VEGFA protein levels were normalized to β-actin and then expressed relative to the corresponding 0 h value in each group. The protein degradation curve was generated based on three independent experiments.

### Animal model and subcutaneous tumor formation assay

C57BL/6 mice (6–8 weeks) were purchased from the Animal Research Center of Hainan Provincial People’s Hospital and housed under standard SPF conditions with free access to food and water (24 ± 1 °C, 50–70% humidity, 12h light/dark cycle). Mice were randomly divided into two groups (n=6 each). All animal experiments were approved by the Animal Ethics Committee of our Hospital and performed in accordance with relevant guidelines. For tumor formation, Hepa1–6 cells stably transduced with sh-NC or sh-TMEM160 were harvested and resuspended in sterile saline. Each mouse received a subcutaneous injection of 5×10^6^ cells into the right flank. Tumor size was measured every 3 days from day 0 to day 15, and tumor volume was calculated as V = (length × width^2^)/2 (21). On day 15, mice were euthanized by CO_2_ inhalation, and tumors were excised, photographed, and weighed.

### Hematoxylin and eosin staining

Paraffin-embedded tumor tissues were cut into 4µm sections. Sections were deparaffinized in xylene and rehydrated through graded ethanol, then stained with hematoxylin followed by eosin. After dehydration and clearing, slides were mounted and examined under a light microscope for histopathological evaluation.

### Immunohistochemistry

Paraffin sections (4µm) were deparaffinized and rehydrated, followed by heat-induced antigen retrieval in citrate buffer (pH 6.0). Endogenous peroxidase activity and nonspecific binding were blocked before overnight incubation at 4 °C with primary antibodies against TMEM160 (Proteintech, 26451-1-AP), VEGFA (Abcam, ab1316), and IFN-γ (Abcam, ab9657). Sections were then incubated with an HRP-conjugated secondary antibody, visualized with DAB, and counterstained with hematoxylin. Images were acquired under a light microscope, and positive staining was quantified in five random fields per section in a blinded manner.

### RT-qPCR

RT-qPCR was performed to quantify TMEM160 mRNA levels. Briefly, total RNA was isolated from cells using RNAiso Plus (Takara, Japan) and reverse-transcribed into cDNA with PrimeScript™ RT reagent kit (Takara, Japan). Quantitative PCR was then carried out using SYBR Premix Ex Taq™ (Takara, Japan) on an ABI PRISM 7300 Sequence Detection System (Applied Biosystems, USA) under the manufacturer-recommended cycling conditions. Primer sequences were: TMEM160 Forward 5’-CTCCGCAAGGCACATGAGA-3’, Reverse 5’-AGACCCACCGCATAGGAGG-3’; GAPDH Forward 5′-CACCCACTCCTCCACCTTTG-3′, Reverse 5′-CCACCACCCTGTTGCTGTAG-3′. GAPDH served as an internal control. The mRNAs’ expression was calculated by the 2-^ΔΔ^Ct method.

### Western blot

Proteins from cells and tumor tissues were extracted with RIPA buffer containing protease inhibitors. Protein concentrations were determined using the Pierce™ BCA Kit (Thermo Scientific). Equal amounts of protein (20μg) were separated by SDS-PAGE, transferred onto PVDF membranes, blocked with 5% milk for 1h, and then incubated overnight at 4 °C with primary antibodies against TMEM160 (1:600, 26451-1-AP, Proteintech), VEGFA (1:1000, ab214424, Abcam), Fibronectin (1:1000, ab2413, Abcam), Vimentin (1:1000, ab8069, Abcam), PD-L1 (1:1000, ab228415, Abcam), PI3K (1:1000, ab191606, Abcam), p-PI3K (1:1000, ab278545, Abcam), AKT (1:10000, ab179463, Abcam), p-AKT (1:1000, ab81283, Abcam), β-actin (1:1000, ab8226, Abcam). Following TBST washes, the membranes were incubated with horseradish peroxidase (HRP)-conjugated goat anti-rabbit IgG secondary antibody (1:1000, ab96899, Abcam) at 37 °C for 45 minutes. Immunoreactive bands were visualized using the Pierce ECL Western Blotting Substrate (Thermo Scientific, Shanghai, China) and captured with a chemiluminescence imaging system.

### Statistical analysis

All statistical analyses were performed using SPSS 26.0 (IBM, Armonk, NY, USA). Mean SD was used to express the measurement data. Comparisons between two groups were performed using a two-tailed Student’s t test. Comparisons among multiple groups were conducted using ANOVA followed by the Tukey *post hoc* test. A chi-square test was used for comparing groups between low and high TMEM160 expression. The K-M curve was utilized for survival analysis. For *in vitro* experiments, all assays were performed in three independent replicates. For *in vivo* experiments, six mice per group were used.

## Results

### TMEM160 was upregulated in HCC and high TMEM160 expression predicted poor prognosis

**Figure 1:**
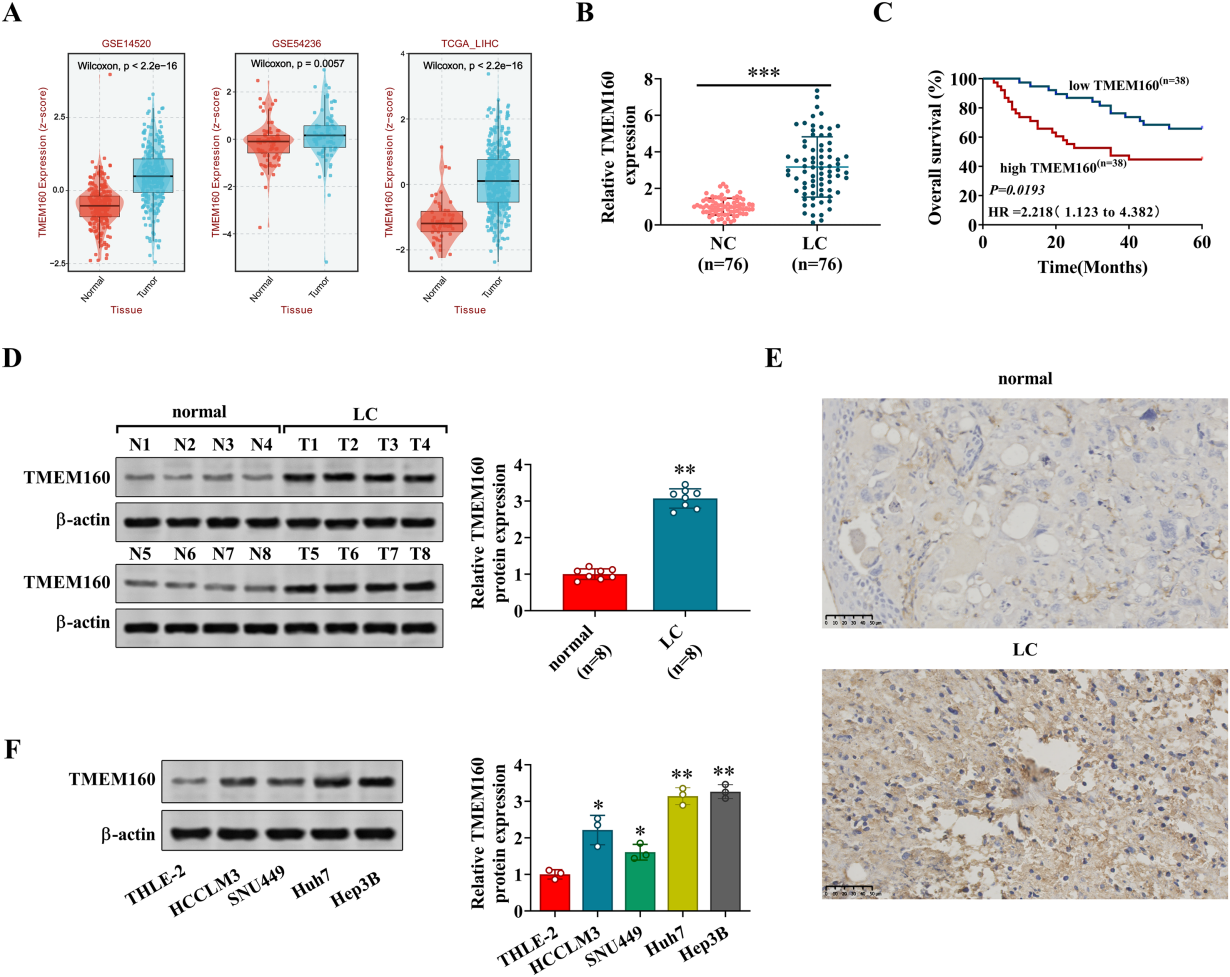
TMEM160 was upregulated in HCC and high TMEM160 expression predicted poor prognosis. **(A)** TMEM160 expression was analyzed in HCC and normal liver tissues in GEO datasets (GSE14520 and GSE54236) and the TCGA_LIHC cohort. **(B)** TMEM160 mRNA expression was detected by RT–qPCR in 76 paired HCC tissues (LC) and adjacent non-tumor tissues (NC), ***P<0.001 vs NC. **(C)** Kaplan–Meier analysis was performed for overall survival in patients with low (n=38) or high (n=38) TMEM160 expression using the median TMEM160 level in LC tissues. **(D)** Western blotting was performed to detect TMEM160 expression in paired HCC tissues and adjacent non-tumor tissues (n=8), **P<0.01 vs normal. **(E)** Immunohistochemical staining was performed to detect TMEM160 expression and localization in HCC tissues and adjacent non-tumor tissues. Scale bar, 50 μm. **(F)** Western blotting was performed to detect TMEM160 expression in THLE-2 and HCC cell lines (HCCLM3, SNU449, Huh7, and Hep3B), *P<0.05, **P<0.01 vs THLE-2. Comparisons between two groups were conducted using Student’s t-test. Comparisons among multiple groups were conducted using one-way analysis of variance (ANOVA) followed by the Tukey *post hoc* test. The log-rank test was used in Kaplan–Meier analysis.

First, we explored the clinical relevance of TMEM160 in HCC. As shown in **Table 1**, compared with the low TMEM160 group, the high TMEM160 group showed a significantly higher proportion of lymph node involvement and advanced TNM stage (P<0.05), whereas age, sex, tumor size, and HBV infection status did not differ between the two groups (P>0.05). Then, TMEM160 expression and its prognostic value were evaluated in HCC patients. TMEM160 expression was markedly increased in HCC tissues in TCGA_LIHC and GEO cohorts (**Figure 1A**). Consistently, RT-qPCR analysis of 76 paired HCC and adjacent non-tumor tissues revealed significantly elevated TMEM160 mRNA expression in tumor tissues (**Figure 1B**; P<0.001). Using the median TMEM160 expression in tumor tissues as the cutoff, and Kaplan–Meier analysis demonstrated that the high TMEM160 group had a significantly poorer overall survival (**Figure 1C**; P<0.05). Protein-level validation further showed that TMEM160 protein expression was elevated in HCC tissues compared with adjacent non-tumor tissues (**Figure 1D**; P<0.01). TMEM160 staining was weak in adjacent non-tumor liver tissues but markedly enhanced in HCC tissues, with positive signals mainly distributed in the cytoplasmic of tumor cells (**Figure 1E**).

**Table 1:** Correlation between TMEM160 expression and the clinical pathological features of 76 liver cancer patients.

Characteristic	All cases	TMEM160 expression		P-value
		Low (n = 38)	High (n = 38)	
Age (years)				0.488
< 55	33	18	15	
≥55	43	20	23	
Gender				0.247
Female	33	19	14	
Male	43	19	24	
Tumour size				0.817
< 5 cm	33	17	16	
≥5 cm	43	21	22	
Lymph node involvement				0.012*
Absent	39	25	14	
Present	37	13	24	
TNM				0.010*
I+II	45	28	17	
III+IV	31	10	21	
HBV infection				0.358
Yes	36	20	16	
No	40	18	22	

A chi-square test was used for comparing groups between low and high TMEM160 expression. *P < 0.05.

Additionally, TMEM160 protein levels were remarkably increased in HCC cell lines (Huh7, Hep3B, HCCLM3, and SNU449) compared with THLE-2 cells (**Figure 1F**; P<0.05). Based on these expression patterns, Huh7 and Hep3B were used for TMEM160 knockdown experiments, whereas SNU449 was used for TMEM160 overexpression experiments in the following studies.

### TMEM160 knockdown inhibited HCC cell proliferation, invasion, and angiogenesis

**Figure 2:**
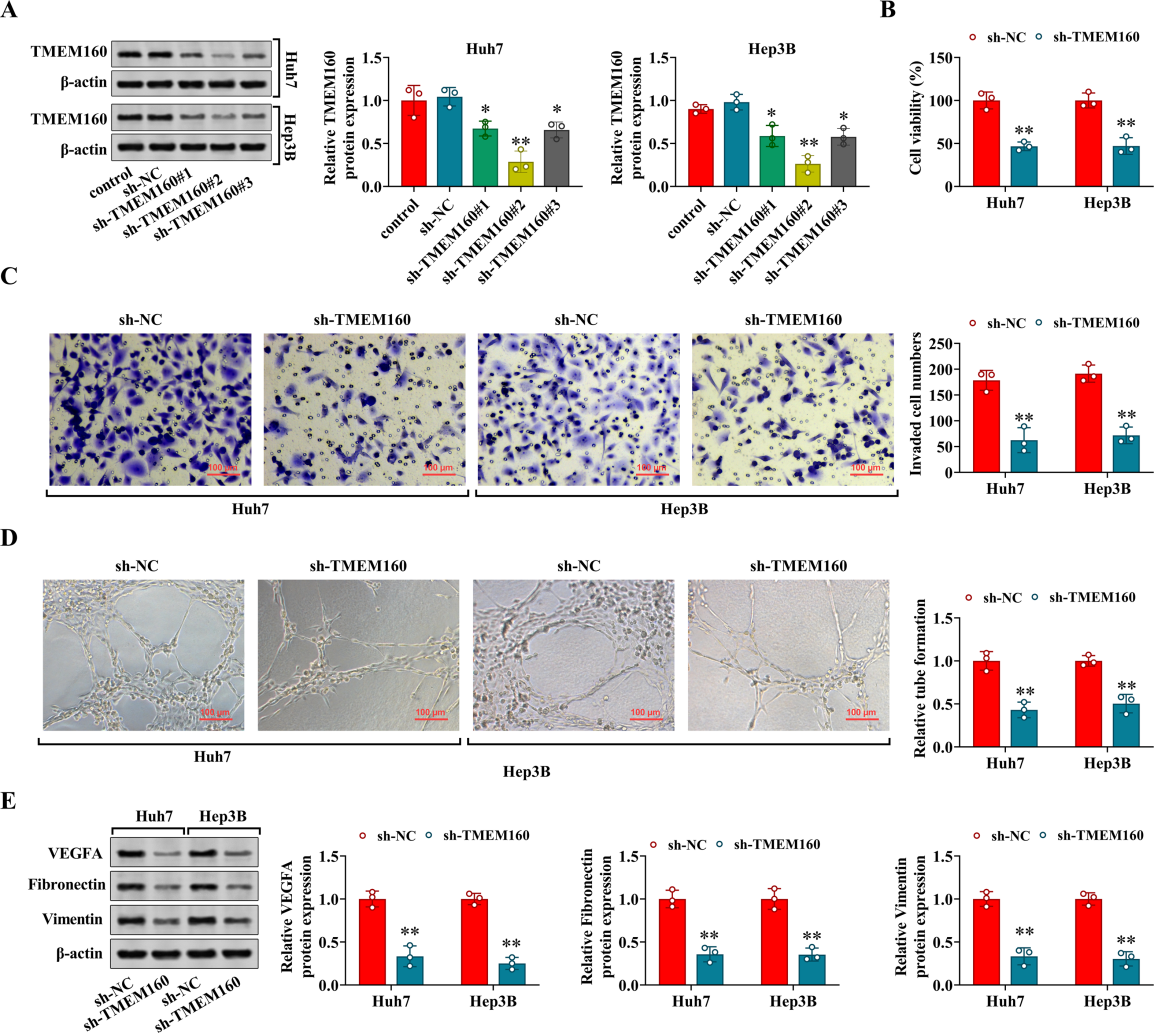
Knockdown of TMEM160 suppressed proliferation, invasion, and angiogenesis of HCC cells. **(A)** Western blotting was performed to evaluate the knockdown efficiency of TMEM160 in Huh7 and Hep3B cells transduced with sh-NC or sh-TMEM160#1/#2/#3. **(B)** Cell viability of Huh7 and Hep3B cells with TMEM160 knockdown was assessed by CCK-8 assay. **(C)** Transwell assays with Matrigel coating were conducted to evaluate the invasive ability of Huh7 and Hep3B cells after TMEM160 knockdown. **(D)** Tube formation assays were performed to assess the angiogenic capacity of HUVECs incubated with conditioned media from sh-NC or sh-TMEM160 Huh7 and Hep3B cells. **(E)** Western blotting was performed to detect the protein expression levels of VEGFA, fibronectin, and vimentin in Huh7 and Hep3B cells with TMEM160 knockdown. Comparisons between two groups were conducted using Student’s t-test. Comparisons among multiple groups were conducted using one-way analysis of variance (ANOVA) followed by the Tukey *post hoc* test. *P<0.05, **P<0.01 vs sh-NC.

To further investigate the effects of TMEM160 on HCC cell behaviors, TMEM160 was knocked down in Huh7 and Hep3B cells. sh-TMEM160#1, sh-TMEM160#2, and sh-TMEM160#3 significantly reduced TMEM160 protein levels in both Huh7 and Hep3B cells (**Figure 2A**; P<0.05), and sh-TMEM160#2, which showed the highest knockdown efficiency, was selected for subsequent experiments. CCK-8 assays showed that TMEM160 knockdown significantly decreased cell viability in both Huh7 and Hep3B cells (**Figure 2B**; P<0.01). Transwell assays with Matrigel showed that the invasive cell numbers were reduced in the sh-TMEM160 group in both cell lines (**Figure 2C**; P<0.01). Conditioned media from TMEM160-silenced Huh7 and Hep3B cells also impaired HUVEC tube formation (**Figure 2D**; P<0.01). Western blotting further showed that TMEM160 knockdown reduced the protein levels of VEGFA, fibronectin, and vimentin in both Huh7 and Hep3B cells (**Figure 2E**; P<0.01). Collectively, these results indicated that TMEM160 knockdown suppressed HCC cell proliferation and invasion and attenuated angiogenic potential.

### TMEM160 knockdown suppressed immune evasion of HCC cells

**Figure 3:**
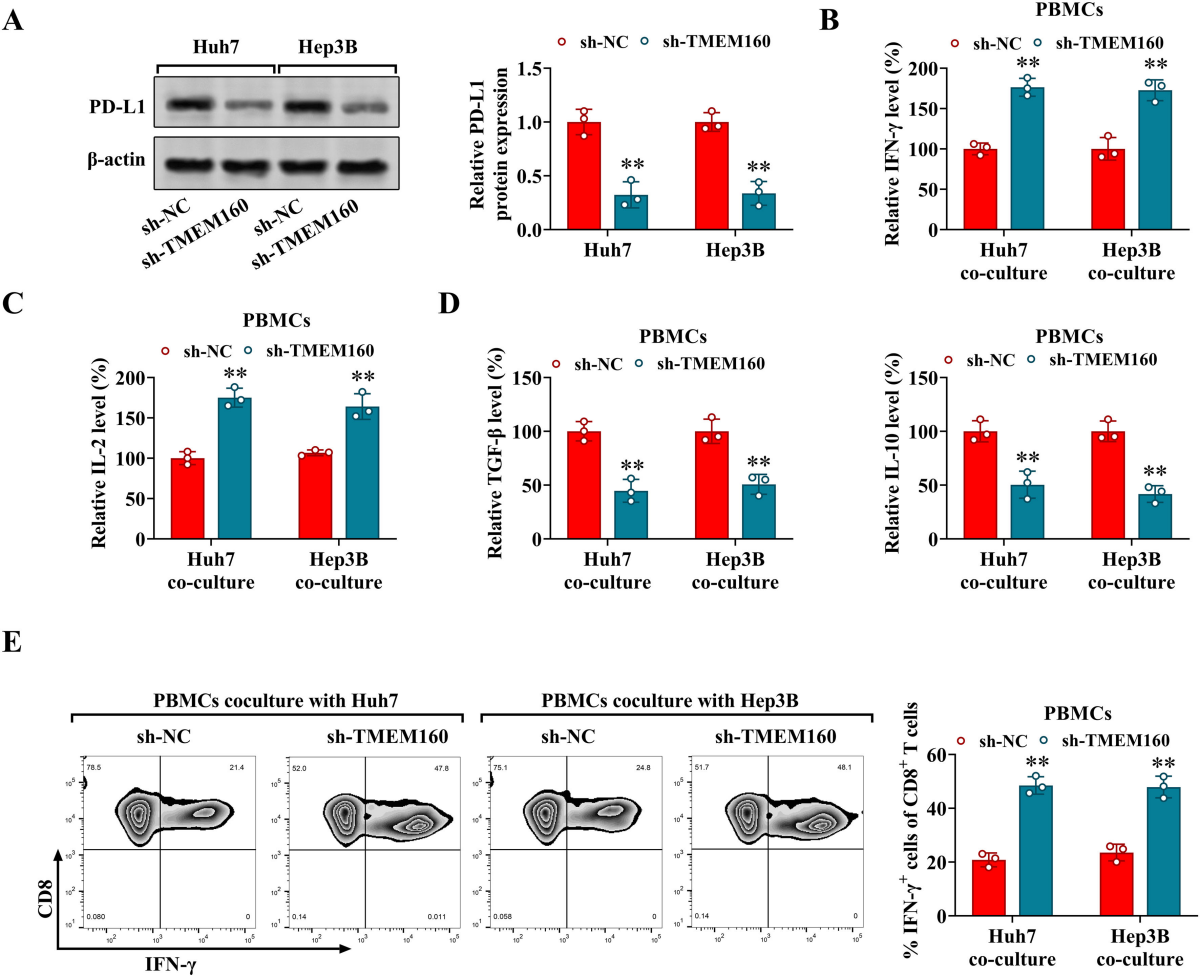
TMEM160 knockdown suppressed immune evasion of HCC cells. **(A)** Western blotting was performed to detect PD-L1 protein expression in Huh7 and Hep3B cells transfected with sh-NC or sh-TMEM160. **(B, C)** ELISA was used to measure IFN-γ and IL-2 levels in PBMCs after co-culture with Huh7 or Hep3B cells transfected with sh-NC or sh-TMEM160. **(D)** ELISA was used to measure TGF-β and IL-10 levels in PBMCs after co-culture with Huh7 or Hep3B cells transfected with sh-NC or sh-TMEM160. **(E)** Flow cytometry was performed to analyze the percentage of CD8^+^IFN-γ^+^ T cells in PBMCs after co-culture with Huh7 or Hep3B cells transfected with sh-NC or sh-TMEM160. Data are presented as mean ± SD. Comparisons between two groups were conducted using Student’s t-test. *P<0.01 vs sh-NC.

To characterize the immunological consequences of TMEM160 knockdown, immune checkpoint expression and PBMC functional responses were examined in the HCC–PBMC co-culture system. TMEM160 knockdown significantly decreased PD-L1 protein levels in both Huh7 and Hep3B cells (**Figure 3A**; P<0.01). According to previous studies (22) indicating that tumor immune evasion is closely associated with alterations in cytokine secretion, representative immune-related cytokines were further analyzed. Moreover, TMEM160 knockdown significantly increased the levels of IFN-γ and IL-2 in PBMCs after co-culture with either Huh7 or Hep3B cells (**Figures 3B, C**; P<0.01). Conversely, the levels of the immunosuppressive cytokines TGF-β and IL-10 were markedly reduced in PBMCs following co-culture with TMEM160-silenced HCC cells (**Figure 3D**; P<0.01). Flow cytometry further demonstrated a significant increase in CD8^+^IFN-γ^+^ T cells in PBMCs co-cultured with Huh7 or Hep3B cells after TMEM160 knockdown (**Figure 3E**; P<0.01). Taken together, these results demonstrated that silencing TMEM160 suppressed immune evasion in HCC cells.

### TMEM160 overexpression promoted HCC cell proliferation, invasion, angiogenesis, and immune evasion

**Figure 4:**
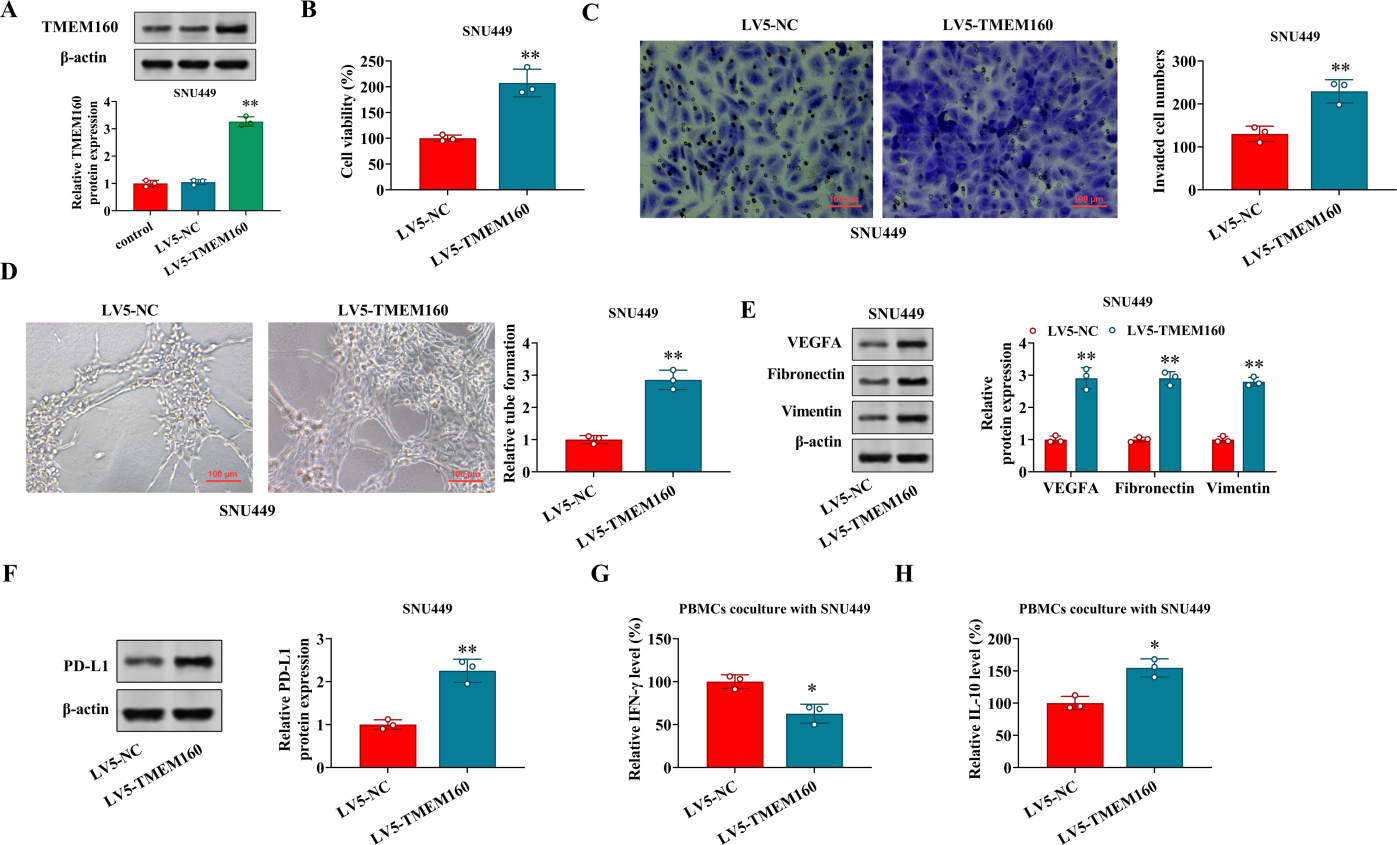
TMEM160 overexpression promoted proliferation, invasion, angiogenesis and immune evasion in HCC cells. **(A)** Western blotting was performed to detect TMEM160 expression in SNU449 cells transduced with control, LV5-NC or LV5-TMEM160. **(B)** Cell viability of SNU449 cells transduced with LV5-NC or LV5-TMEM160 was assessed by CCK-8 assay. **(C)** Transwell invasion assay with Matrigel was performed to evaluate the invasive ability of SNU449 cells transduced with LV5-NC or LV5-TMEM160. **(D)** Tube formation assay was conducted to assess the angiogenic capacity of HUVECs cultured with conditioned medium from SNU449 cells transduced with LV5-NC or LV5-TMEM160. **(E)** Western blotting was performed to detect VEGFA, fibronectin and vimentin expression in SNU449 cells transduced with LV5-NC or LV5-TMEM160. **(F)** Western blotting was performed to detect PD-L1 expression in SNU449 cells transduced with LV5-NC or LV5-TMEM160. ELISA was performed to detect IFN-γ **(G)** and IL-10 **(H)** levels in PBMCs after co-culture with SNU449 cells transduced with LV5-NC or LV5-TMEM160. Comparisons between two groups were conducted using Student’s t-test. *P<0.05, **P<0.01 vs LV5-NC.

To assess the effects of TMEM160 overexpression, SNU449 cells stably transfected with LV5-TMEM160 were subjected to functional assays. TMEM160 protein expression was significantly increased in the LV5-TMEM160 group (**Figure 4A**; P<0.01). CCK-8 assays showed that TMEM160 overexpression significantly enhanced cell viability (**Figure 4B**; P<0.01). Consistently, Matrigel-coated Transwell assays revealed that TMEM160 overexpression significantly increased invasive ability in SNU449 cells (**Figure 4C**; P<0.01). In addition, conditioned medium from LV5-TMEM160 cells significantly promoted HUVEC tube formation relative to LV5-NC (**Figure 4D**; P<0.01). In addition, VEGFA, Fibronectin, and Vimentin protein levels were significantly upregulated upon TMEM160 overexpression (**Figure 4E**; P<0.01). Moreover, PD-L1 protein expression was remarkably elevated in the LV5-TMEM160 group (**Figure 4F**; P<0.01). In the PBMC co-culture system, TMEM160 overexpression reduced IFN-γ levels and increased IL-10 levels significantly (**Figures 4G, H**; both P<0.05). Overall, these results suggested that TMEM160 overexpression facilitated HCC aggressiveness, enhanced pro-angiogenic potential, and strengthened immune evasion.

### TMEM160 regulated the VEGFA/PI3K/AKT signaling pathway in HCC cells

**Figure 5:**
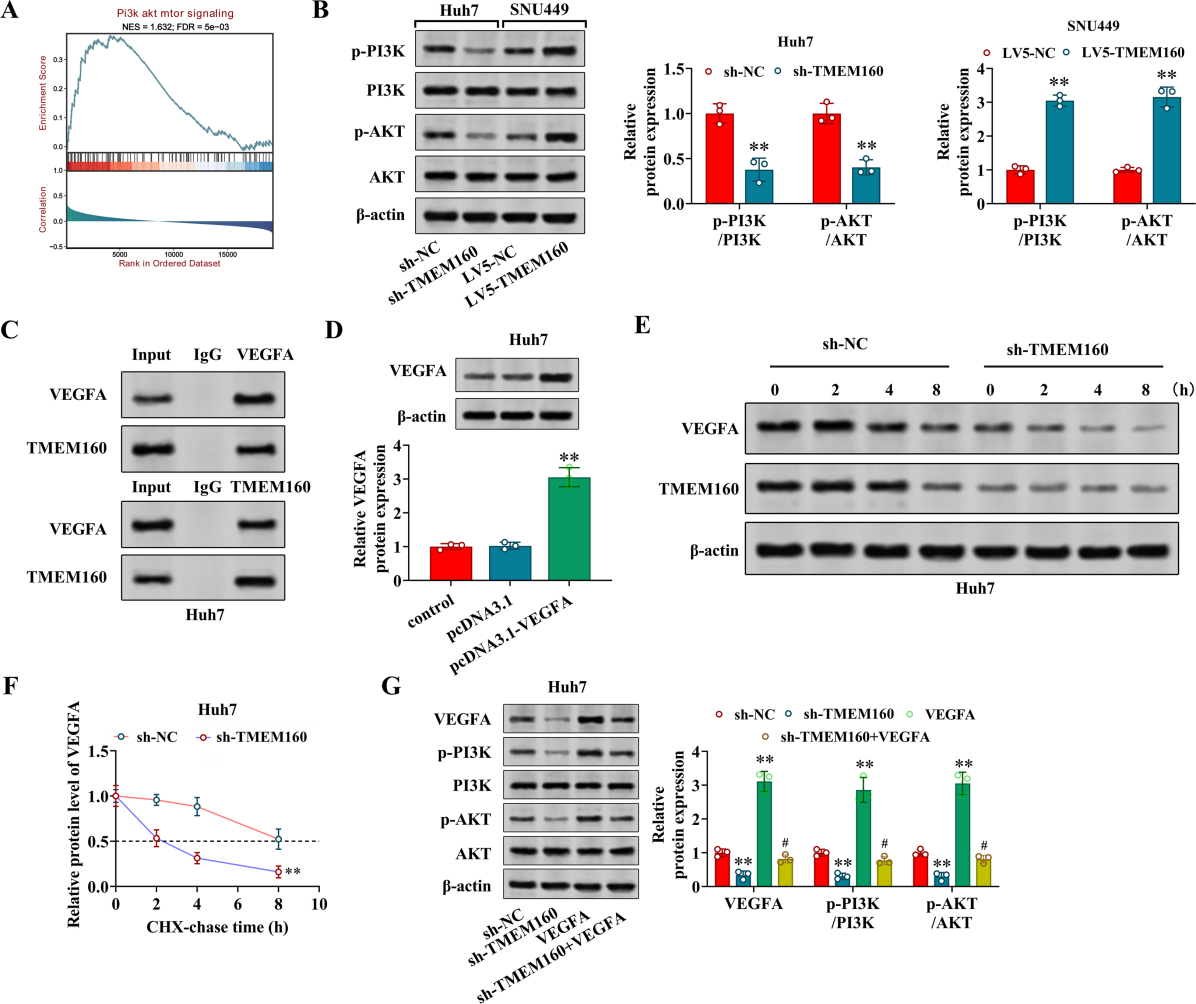
TMEM160 regulated the VEGFA/PI3K/AKT signaling pathway in HCC cells. **(A)** Gene set enrichment analysis (GSEA) based on the BEST application revealed a significant enrichment of the PI3K/AKT signaling pathway associated with TMEM160 expression. **(B)** Western blotting was performed to detect p-PI3K, PI3K, p-AKT, and AKT expression in Huh7 cells (**P<0.01 vs sh-NC) and SNU449 cells (**P<0.01 vs LV5-NC). **(C)** Reciprocal Co-IP assays were performed to examine the association between TMEM160 and VEGFA in Huh7 cells. **(D)** Western blotting detection of VEGFA expression in Huh7 cells transfected with pcDNA3.1-VEGFA, **P<0.01 vs pcDNA3.1. **(E)** CHX chase assay was performed to detect VEGFA protein degradation in Huh7 cells transfected with sh-NC or sh-TMEM160. **(F)** Quantitative analysis of VEGFA protein levels after CHX treatment, **P<0.01 vs sh-NC. **(G)** Western blotting analysis of VEGFA, p-PI3K, PI3K, p-AKT, and AKT expression in Huh7 cells under different conditions, **P<0.01 vs sh-NC; ^#^P<0.05 vs sh-TMEM160. Data were presented as mean ± SD. Comparisons between two groups were conducted using Student’s t-test. Comparisons among multiple groups were performed using one-way ANOVA followed by Tukey’s *post hoc* test.

Based on the findings in **Figures 2**, **4**, TMEM160 was shown to regulate VEGFA expression in HCC cells. GSEA analysis using the BEST application (https://www.gsea-msigdb.org/gsea/index.jsp) further indicated an association between TMEM160 and the PI3K/AKT signaling pathway (**Figure 5A**). Previous studies have reported that the VEGFA/PI3K/AKT axis is critically involved in tumor malignant progression, including proliferation, invasion, and angiogenesis (23–25), and VEGFA has also been closely linked to tumor immune evasion (26). Therefore, we hypothesized that TMEM160 may influence malignant progression and immune escape in HCC by modulating the VEGFA/PI3K/AKT pathway.

In Huh7 cells, TMEM160 silencing reduced the phosphorylation levels of PI3K and AKT (**Figure 5B**; P<0.01). In SNU449 cells, TMEM160 overexpression increased p-PI3K/PI3K and p-AKT/AKT ratio (**Figure 5B**; P<0.01). To further examine whether TMEM160 was associated with VEGFA, reciprocal Co-IP assays were performed in Huh7 cells. When VEGFA was immunoprecipitated, TMEM160 was detected in the VEGFA-immunoprecipitated complex. In addition, when TMEM160 was immunoprecipitated, VEGFA was detected in the TMEM160-immunoprecipitated complex (**Figure 5C**). Western blotting further showed that pcDNA3.1 VEGFA transfection increased VEGFA protein expression in Huh7 cells (**Figure 5D**, P<0.01). To determine whether TMEM160 affected VEGFA protein stability, a CHX chase assay was performed in Huh7 cells. VEGFA protein levels declined more rapidly after TMEM160 knockdown than in sh-NC cells at the indicated time points after CHX treatment (**Figure 5E**). Quantitative analysis of the remaining VEGFA protein showed that TMEM160 knockdown shortened the apparent half-life of VEGFA and significantly reduced VEGFA protein levels (**Figure 5F**; P<0.01), suggesting that TMEM160 knockdown reduced VEGFA protein stability. Moreover, TMEM160 knockdown decreased VEGFA expression as well as the p-PI3K/PI3K and p-AKT/AKT ratios, whereas VEGFA overexpression markedly increased these levels. Importantly, compared with the sh-TMEM160 group, co-transfection of VEGFA in TMEM160-silenced cells significantly enhanced VEGFA expression and the p-PI3K/PI3K and p-AKT/AKT ratios (**Figure 5G**; P<0.05). These results suggested that TMEM160 was involved in the regulation of the VEGFA/PI3K/AKT signaling pathway in HCC cells.

### TMEM160 regulated HCC cell proliferation, invasion, angiogenesis, and immune evasion through the VEGFA/PI3K/AKT axis

**Figure 6:**
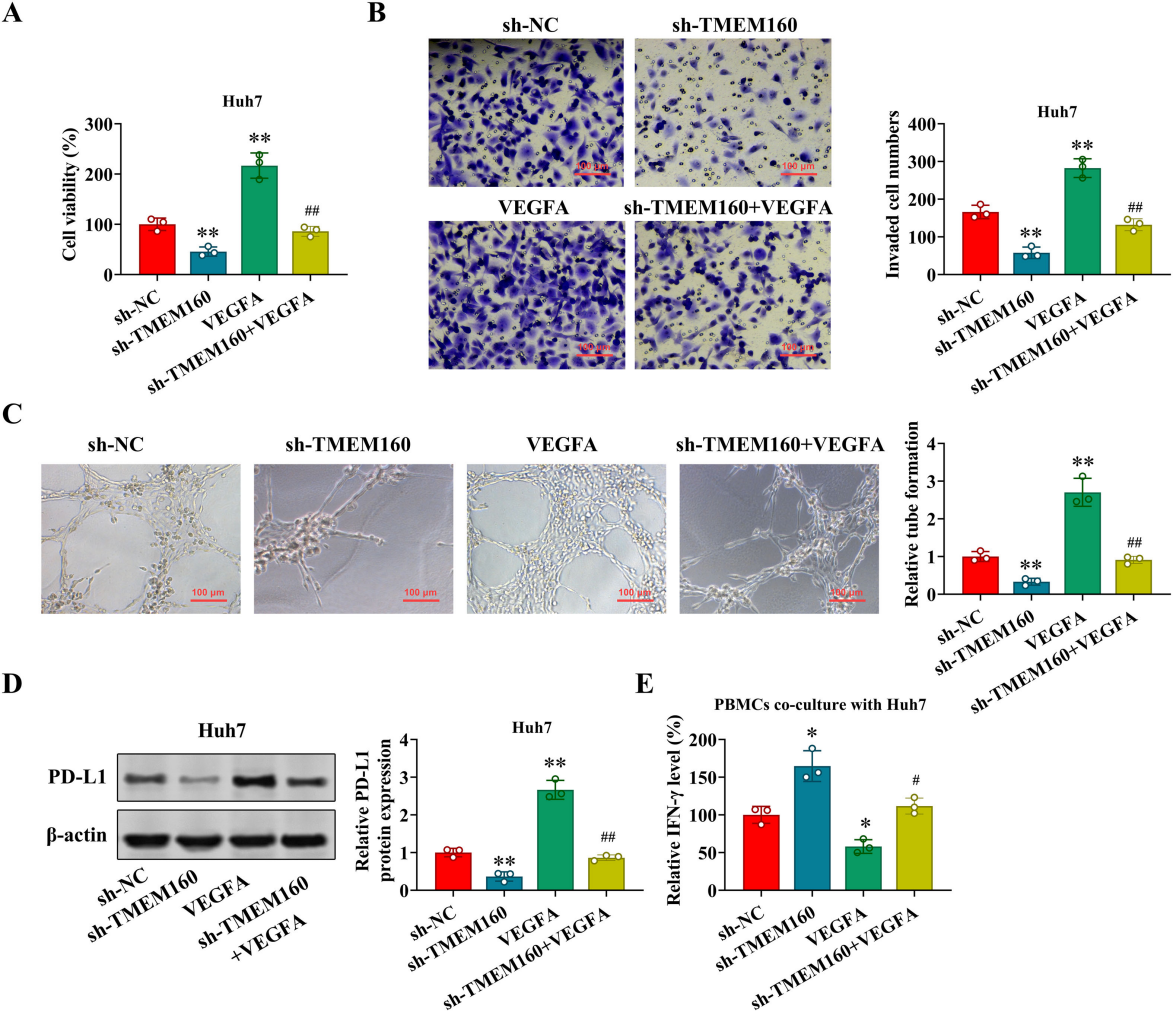
TMEM160 regulated proliferation, invasion, angiogenesis, and immune evasion of HCC cells through VEGFA. **(A)** Cell viability of Huh7 cells was assessed by CCK-8 assay. **(B)** Matrigel-coated Transwell assays were performed to evaluate the invasive ability of Huh7 cells in the indicated groups. **(C)** Tube formation assays were conducted using conditioned medium from Huh7 cells in different groups to assess angiogenic capacity. **(D)** Western blotting was performed to detect PD-L1 protein expression in Huh7 cells from the indicated groups. **(E)** ELISA was used to measure IFN-γ levels in PBMCs co-cultured with Huh7 cells from different groups. Comparisons among multiple groups were conducted using one-way analysis of variance followed by the Tukey *post hoc* test. *P<0.05, **P<0.01 vs sh-NC; ^#^P<0.05, ^##^P<0.01 vs sh-TMEM160.

Rescue assays were conducted in Huh7 cells by combining TMEM160 knockdown with VEGFA overexpression. In CCK-8 assays, TMEM160 knockdown reduced cell viability, whereas VEGFA overexpression increased cell viability. Co-transfection of VEGFA in TMEM160-silenced cells significantly increased cell viability compared with the sh-TMEM160 group (**Figure 6A**; P<0.01). TMEM160 knockdown reduced cell invasion, VEGFA overexpression enhanced invasion, and co-transfection of VEGFA in TMEM160-silenced cells remarkably elevated the number of invaded cells (**Figure 6B**; P<0.01). Similarly, conditioned medium from TMEM160-silenced cells yielded markedly fewer tube-like structures, whereas VEGFA overexpression enhanced HUVEC tube formation, and VEGFA co-transfection significantly increased tube formation relative to the sh-TMEM160 group (**Figure 6C**; P<0.01). Consistently, as shown in **Figure 6D**; PD-L1 expression decreased with TMEM160 knockdown and increased after VEGFA overexpression, and VEGFA co-transfection significantly elevated PD-L1 levels (P<0.01). Additionally, IFN-γ secretion increased after TMEM160 knockdown and decreased upon VEGFA overexpression, and VEGFA co-transfection significantly reduced IFN-γ levels (**Figure 6E**; P<0.05). Taken together, these findings suggested that TMEM160 modulated proliferation, invasion, angiogenesis, and immune evasion in HCC cells by regulating the VEGFA/PI3K/AKT signaling axis.

### TMEM160 knockdown suppressed *in vivo* tumor growth and immune evasion in HCC

**Figure 7:**
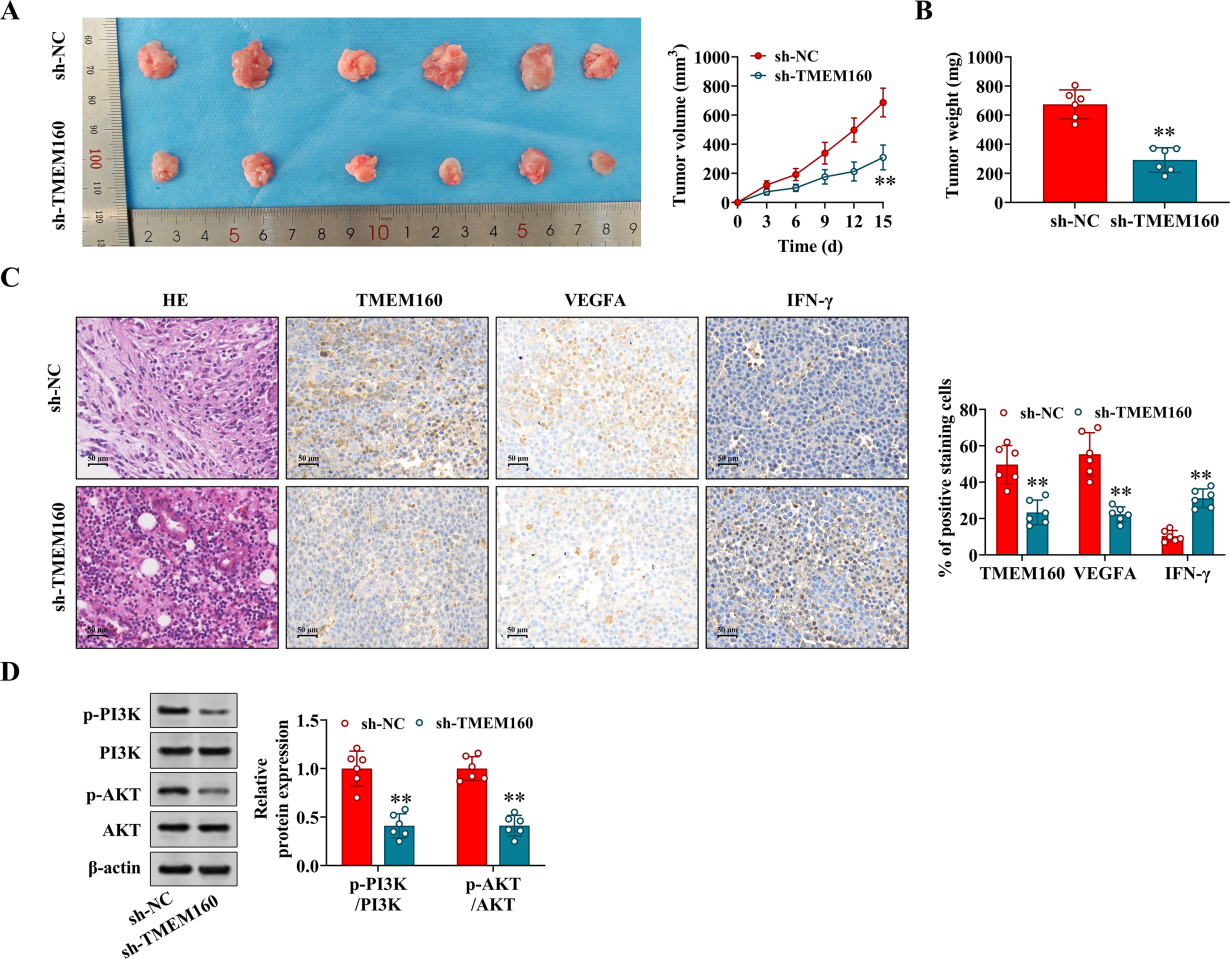
TMEM160 knockdown suppressed tumor growth and immune evasion *in vivo.* **(A)** Representative images of subcutaneous tumors formed by sh-NC or sh-TMEM160 Hepa 1–6 cells in mice and tumor volume growth curves. **(B)** Endpoint tumor weights in the sh-NC and sh-TMEM160 groups. **(C)** HE staining and IHC staining of TMEM160, VEGFA, and IFN-γ in tumor tissues from the sh-NC and sh-TMEM160 groups, with quantification of the percentage of positive cells (scale bar, 50 μm). **(D)** Western blotting detection of p-PI3K, PI3K, p-AKT, and AKT in tumor tissues from the sh-NC and sh-TMEM160 groups. Data are presented as mean ± SD. Continuous data between two groups were compared using Student’s t-test. **P<0.01 vs sh-NC.

**Figure 8:**
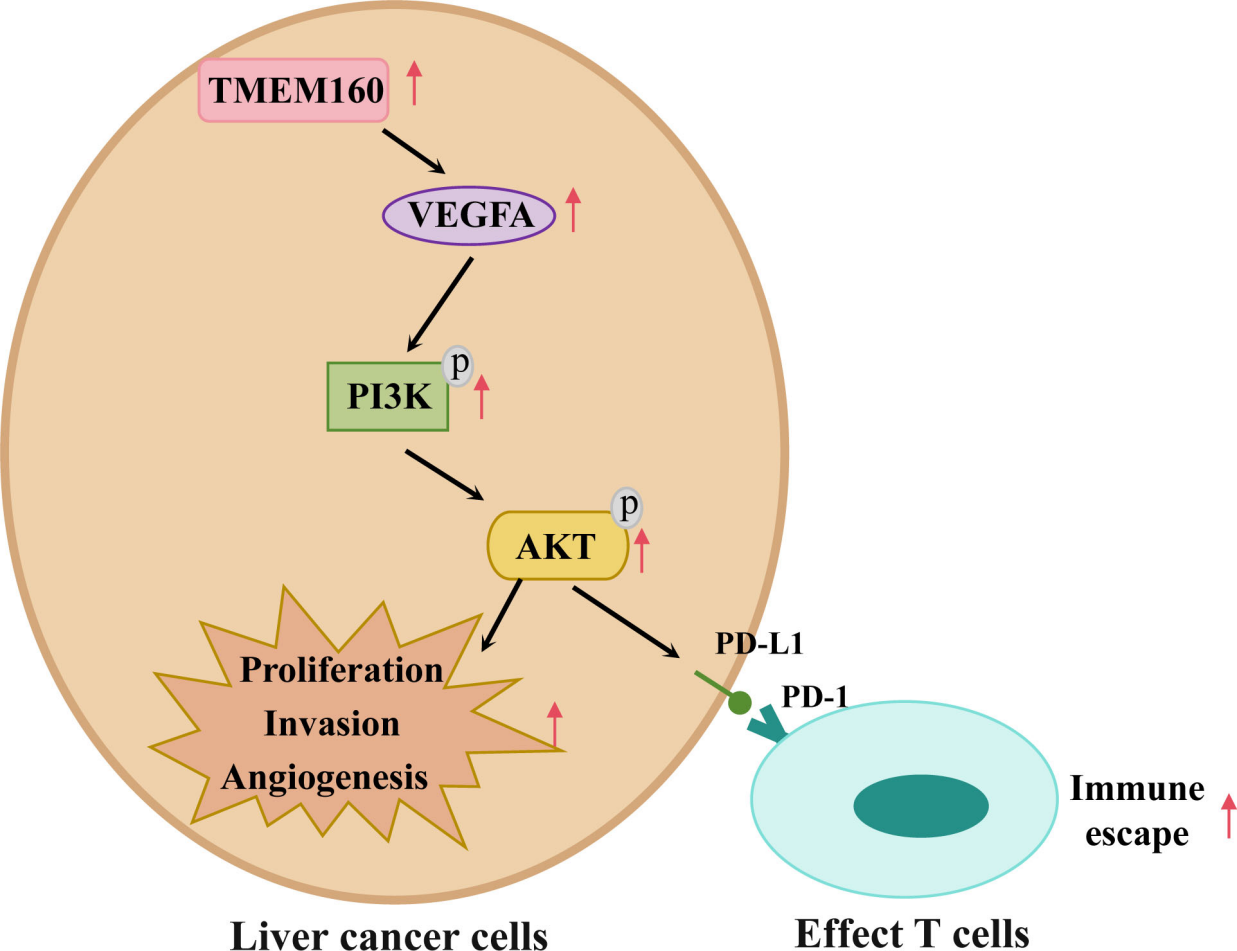
Schematic illustration of the proposed mechanism by which TMEM160 promotes malignant progression and immune escape in hepatocellular carcinoma. TMEM160 upregulation enhances VEGFA expression, leading to activation of the PI3K/AKT signaling pathway, as reflected by increased phosphorylation of PI3K and AKT. Activation of this axis promotes tumor cell proliferation, invasion, and angiogenesis. Concurrently, AKT signaling upregulates PD-L1 expression on liver cancer cells, facilitating PD-1/PD-L1–mediated suppression of effector T-cell function and promoting immune escape.

Finally, we confirmed the inhibitory effect of TMEM160 knockdown on tumor growth and immune evasion in a subcutaneous murine HCC model. Representative images of subcutaneous tumors and the tumor growth curve showed that tumors in the sh-TMEM160 group grew markedly slower and exhibited significantly smaller tumor volumes than those in the sh-NC group (**Figure 7A**; P<0.01). Consistently, tumor weight at the endpoint was significantly reduced in the sh-TMEM160 group (**Figure 7B**; P<0.01). HE staining and immunohistochemical analysis demonstrated that the proportions of TMEM160- and VEGFA-positive cells were significantly reduced, whereas the proportion of IFN-γ–positive cells was markedly increased in tumors derived from sh-TMEM160 cells (**Figure 7C**; P<0.01). Moreover, Western blotting revealed that the p-PI3K/PI3K and p-AKT/AKT ratios were significantly decreased after TMEM160 knockdown (**Figure 7D**; P<0.01). Overall, these results indicated that TMEM160 knockdown inhibited HCC growth *in vivo* and attenuated immune evasion, which was accompanied by suppression of the VEGFA/PI3K/AKT pathway. Graphical abstract was shown in **Figure 8**.

## Discussion

Despite ongoing advances in locoregional and systemic therapies, clinical outcomes for HCC remain limited by persistent disease aggressiveness and marked variability in treatment responsiveness across patients (27, 28). In this context, identifying molecular determinants that couple malignant progression with therapy-relevant phenotypes remains an important priority. In this study, we found that TMEM160 is upregulated in HCC and associated with poorer survival, and our functional and mechanistic data support a role for TMEM160 in promoting malignant phenotypes in concert with activation of the VEGFA/PI3K/AKT axis. These findings provide a rationale to further consider the clinical relevance of TMEM160 and how our observations relate to emerging evidence from other cancer contexts.

Current evidence on TMEM160 remains limited, yet emerging studies suggest that it may exert pro-tumorigenic functions across multiple cancer types and relate to therapeutic resistance or immunosuppressive phenotypes. In colorectal cancer, TMEM160 interacts with PD-L1 and inhibit its ubiquitination-dependent degradation, thereby stabilizing PD-L1 expression and promoting tumor progression, radioresistance, and immune evasion, and clinical specimens further showed that high TMEM160 expression coincided with elevated PD-L1 levels and poorer prognosis (11). In gastric cancer, TMEM160 was shown to activate the TRIM37–KEAP1/NRF2 axis, and overexpression of TMEM160 was identified as an independent predictor of unfavorable outcomes (29). Beyond these reports, studies in lung and cervical cancer models demonstrated that TMEM160 knockdown reduces tumor cell proliferation and suppresses xenograft growth, supporting a potential role for TMEM160 in cancer progression through multiple biological processes (12). Consistent with these cross-cancer clues, we observed that the expression of TMEM160was increased in HCC tissues and that higher expression of TMEM160 was associated with worse survival, providing clinical correlative support for its potential importance in HCC. Building on these observations, we further evaluated the impact of TMEM160 on canonical malignant phenotypes in HCC models.

HCC progression relies on sustained proliferation, acquisition of invasive and migratory capacity, and the formation of new blood vessels, and angiogenesis is widely regarded as a fundamental biological basis supporting tumor growth and disease advancement (30, 31). Among pro-angiogenic factors, VEGFA has repeatedly been positioned as a key driver in HCC. Clinical studies have shown that VEGF expression in HCC tissues is significantly correlated with increased microvessel density and is associated with aggressive features such as vascular invasion or metastasis (32). Mechanistic and functional studies further demonstrate that upstream transcriptional programs that enhance VEGFA expression and secretion in HCC cells can promote endothelial cell migration and tube formation and thereby strengthen angiogenic potential, supporting a direct functional contribution of tumor-derived VEGFA to angiogenesis (33). Consistently, suppression of VEGFA-related expression has been reported to reduce microvessel density in xenografts and attenuate metastasis-associated phenotypes, suggesting that VEGFA is not only a marker of angiogenesis but also an effector linking angiogenesis to invasive progression (34). In line with these observations, we found that TMEM160 knockdown or overexpression correspondingly altered HCC cell proliferation and invasion and modulated endothelial tube formation induced by tumor-conditioned media, accompanied by concordant changes in VEGFA levels. In a xenograft model, TMEM160 silencing similarly restrained tumor growth and was associated with decreased VEGFA in tumor tissues, further supporting a connection between TMEM160 and angiogenesis-related malignant phenotypes. Thus, next focused on VEGFA-associated downstream signaling to clarify how TMEM160 may mediate these effects.

The PI3K/AKT pathway has been repeatedly implicated in key processes underlying HCC progression, including tumor cell survival, invasive growth, and metabolic adaptation, and it remains an active focus of therapeutic exploration (35, 36). Prior HCC studies also place VEGFA and PI3K/AKT activation within a convergent pro-angiogenic context. In this context, KLF8 has been reported to increase VEGFA and promote angiogenesis through FAK-dependent activation of PI3K/AKT with downstream regulation of HIF-1α (37). Tumor-derived CD147-positive small extracellular vesicles have also been shown to activate PI3K/Akt signaling and elevate VEGFA, thereby enhancing endothelial angiogenic responses (38). In addition, FBXO22 has been linked to an AKT-driven increase in HIF-1α and VEGF-A that supports angiogenesis and metastasis-related phenotypes in HCC models (39). Consistent with this established framework, our GSEA suggested enrichment of PI3K/AKT-related signaling in association with TMEM160, and immunoblotting showed that TMEM160 knockdown reduced p-PI3K and p-AKT, whereas TMEM160 overexpression produced the opposite pattern. Notably, VEGFA overexpression increased PI3K/AKT phosphorylation and partially rescued the inhibitory effects caused by TMEM160 silencing, supporting a functional role of the VEGFA–PI3K/AKT axis in TMEM160-mediated malignant phenotypes. Moreover, given the importance of PD-L1 in immunosuppression and therapeutic response in HCC (40), the observation that TMEM160 modulation was accompanied by changes in PD-L1 expression and PBMC co-culture immune readouts suggests that TMEM160-associated signaling may parallel an immunosuppressive phenotype, although confirmation in more physiologically relevant *in vivo* settings is still warranted.

Although direct evidence regarding TMEM160-mediated immune evasion in HCC remains limited, related studies support the potential involvement of TMEM proteins in tumor immune regulation. In colorectal cancer, TMEM160 was reported to interact with PD-L1 and stabilize PD-L1 protein expression by inhibiting SPOP-mediated ubiquitination-dependent degradation, thereby promoting tumor immune evasion and radioresistance (11). In HCC, several TMEM family members have recently been linked to the immune microenvironment. TMEM101 expression was reported to be associated with immune infiltration and prognosis in HCC, with bioinformatic analyses suggesting that its pro-tumor effect may be related to reduced anti-tumor immune cell infiltration and increased M0 macrophage infiltration (41). Another HCC study identified TMEM115 as an oncogenic and immunological biomarker; TMEM115 expression was associated with immune cell infiltration, immune checkpoint-related features, and potential immunotherapy response (42). Beyond HCC, TMEM176B has been characterized as an immune-regulatory transmembrane protein, and targeting TMEM176B was shown to enhance antitumor immunity and augment the efficacy of immune checkpoint blockade by unleashing inflammasome activation (43, 44). These findings suggest that TMEM proteins may participate in shaping the tumor immune microenvironment. In this context, our findings that TMEM160 knockdown decreased PD-L1 expression, enhanced IFN-γ and IL-2 secretion, reduced IL-10 and TGF-β secretion, and increased CD8^+^IFN-γ^+^ T cells provide preliminary evidence that TMEM160 may contribute to immune evasion in HCC.

Increasing evidence suggests that TMEM family members can regulate tumor progression through diverse and context-dependent mechanisms, and many TMEM proteins remain incompletely characterized in terms of their downstream molecular targets and regulatory modes (8). Notably, a recent study in colorectal cancer reported that TMEM160 interacts with PD-L1 and stabilizes PD-L1 protein expression by competing with SPOP, thereby inhibiting SPOP-mediated ubiquitination-dependent degradation of PD-L1 (11). In addition, other TMEM proteins have also been implicated in protein stability regulation. TMEM100 was shown to promote HIF-1α degradation through the ubiquitin–proteasome pathway, thereby reducing VEGF release and suppressing angiogenesis-related phenotypes (45), whereas TMEM127 was reported to promote RET ubiquitination, subcellular positioning, and degradation (46). These findings suggest that TMEM160 may potentially regulate VEGFA or VEGFA-related signaling through protein stability, ubiquitination-dependent degradation, or other post-translational mechanisms. Meanwhile, VEGFA expression can also be regulated at the transcriptional level through PI3K/AKT/HIF-1α-related signaling in HCC. KLF8 was reported to induce VEGFA promoter activity and regulate HIF-1α expression through FAK-dependent PI3K/AKT activation in HCC 37, and miR-26a was shown to inhibit HCC angiogenesis by downregulating the PIK3C2α/Akt/HIF-1α/VEGFA pathway (47). Therefore, although our Co-IP, CHX chase, and rescue experiments support an association between TMEM160 and VEGFA-related PI3K/AKT signaling and indicate that TMEM160 contributes to VEGFA protein stability, further studies are still needed to determine the exact degradation pathway, VEGFA ubiquitination pattern, and potential upstream transcriptional regulators of VEGFA.

## Conclusions

Our result indicated that TMEM160 promoted HCC cell proliferation and invasion and was accompanied by immune escape–related changes. These effects were suggested to be mediated, at least in part, by TMEM160-dependent regulation of the VEGFA-driven PI3K/AKT pathway. Collectively, our findings indicated that TMEM160 acted upstream of the VEGFA/PI3K/AKT axis to facilitate malignant progression and immune suppression associated phenotypes in HCC, providing a mechanistic rationale for considering TMEM160 as a potential therapeutic target.

Several limitations should be acknowledged. The clinical analyses were based on a single-center cohort of 76 resected HCC cases collected between 2019 and 2020, which may constrain generalizability and warrants validation in larger, independent, multi-center populations. Functional experiments relied primarily on established cell lines and a subcutaneous murine tumor model, which cannot fully recapitulate intrahepatic tumor biology, vascular context, and metastatic behavior in patients. The angiogenesis and immune-related observations were supported by conditioned-medium tube formation and PBMC co-culture readouts together with limited tumor-tissue markers, and more comprehensive *in vivo* and clinical immune profiling will be needed to confirm relevance to the native HCC microenvironment and treatment response. Mechanistically, although Co-IP assays, CHX chase experiments, VEGFA rescue experiments, and concordant PI3K/AKT phosphorylation changes support a functional association between TMEM160 and VEGFA-related PI3K/AKT signaling, the direct molecular basis by which TMEM160 regulates VEGFA was not fully delineated. In particular, although the CHX chase assay indicated that TMEM160 knockdown accelerated VEGFA protein degradation, the exact degradation pathway, ubiquitination pattern, responsible E3 ligase machinery, transcriptional contribution, and upstream regulators such as HIF-1α remain to be clarified in future studies.

## Data Availability

In this study, RNA-seq expression data and corresponding clinical information for TCGA_LIHC were obtained from The Cancer Genome Atlas through the GDC Data Portal (https://portal.gdc.cancer.gov/). GEO datasets GSE14520 and GSE54236 were obtained from the NCBI Gene Expression Omnibus database (https://www.ncbi.nlm.nih.gov/geo/). For pathway enrichment analysis, gene set enrichment analysis (GSEA) was performed using the BEST application (Biomarker Exploration for Solid Tumors, https://www.gsea-msigdb.org/gsea/index.jsp) to explore signaling pathways associated with TMEM160 expression in HCC.

## References

[1] Bray F Laversanne M Sung H Ferlay J Siegel RL Soerjomataram I . Global cancer statistics 2022: GLOBOCAN estimates of incidence and mortality worldwide for 36 cancers in 185 countries. CA Cancer J Clin. (2024) 74:229–63. doi: 10.3322/caac.21834 38572751

[2] Li Q Cao M Lei L Yang F Li H Yan X . Burden of liver cancer: From epidemiology to prevention. Chin J Cancer Res. (2022) 34:554–66. doi: 10.21147/j.issn.1000-9604.2022.06.02 36714347 PMC9829497

[3] Galle PR Forner A Llovet JM Mazzaferro V Piscaglia F Raoul J-L . EASL clinical practice guidelines: Management of hepatocellular carcinoma. J Hepatol. (2018) 69:182–236. doi: 10.1016/j.jhep.2018.03.019 29628281

[4] Singal AG Llovet JM Yarchoan M Mehta N Heimbach JK Dawson LA . AASLD practice guidance on prevention, diagnosis, and treatment of hepatocellular carcinoma. Hepatology. (2023) 78:1922–65. doi: 10.1097/hep.0000000000000466 37199193 PMC10663390

[5] Finn RS Qin S Ikeda M Galle PR Ducreux M Kim TY . Atezolizumab plus bevacizumab in unresectable hepatocellular carcinoma. N Engl J Med. (2020) 382:1894–905. doi: 10.1056/NEJMoa1915745 32402160

[6] Abou-Alfa GK Lau G Kudo M Chan SL Kelley RK Furuse J . Tremelimumab plus durvalumab in unresectable hepatocellular carcinoma. NEJM Evid. (2022) 1:EVIDoa2100070. doi: 10.1056/EVIDoa2100070 38319892

[7] Ntellas P Chau I . Updates on systemic therapy for hepatocellular carcinoma. Am Soc Clin Oncol Educ Book. (2024) 44:e430028. doi: 10.1200/edbk_430028 38175973

[8] Herrera-Quiterio GA Encarnación-Guevara S . The transmembrane proteins (TMEM) and their role in cell proliferation, migration, invasion, and epithelial-mesenchymal transition in cancer. Front Oncol. (2023) 13:1244740. doi: 10.3389/fonc.2023.1244740 37936608 PMC10627164

[9] Yamashita K Haraguchi M Yano M . Knockdown of TMEM160 leads to an increase in reactive oxygen species generation and the induction of the mitochondrial unfolded protein response. FEBS Open Bio. (2022) 12:2179–90. doi: 10.1002/2211-5463.13496 36217717 PMC9714381

[10] Akmeriç EB Gerhardt H . Blood flow meets mitophagy. J Cell Biol. (2022) 221:e202206033. doi: 10.1083/jcb.202206033 35727256 PMC9213090

[11] Dai X Wu Z Ruan R Chen J Huang C Lei W . TMEM160 promotes tumor immune evasion and radiotherapy resistance via PD-L1 binding in colorectal cancer. Cell Commun Signal. (2024) 22:168. doi: 10.1186/s12964-024-01541-w 38454413 PMC10921666

[12] Herrera-Quiterio GA Valencia-González HA de la Cruz-López KG Fernández-Coto DL Gil J Marko-Varga G . TMEM160 promotes tumor growth in lung adenocarcinoma and cervical adenocarcinoma cell lines. Int J Mol Sci. (2025) 26:1097. doi: 10.3390/ijms26031097 39940865 PMC11816668

[13] Yao C Wu S Kong J Sun Y Bai Y Zhu R . Angiogenesis in hepatocellular carcinoma: Mechanisms and anti-angiogenic therapies. Cancer Biol Med. (2023) 20:25–43. doi: 10.20892/j.issn.2095-3941.2022.0449 36647777 PMC9843448

[14] Zhu XD Tang ZY Sun HC . Targeting angiogenesis for liver cancer: Past, present, and future. Genes Dis. (2020) 7:328–35. doi: 10.1016/j.gendis.2020.03.010 32884987 PMC7452391

[15] Finn RS Zhu AX . Targeting angiogenesis in hepatocellular carcinoma: Focus on VEGF and bevacizumab. Expert Rev Anticancer Ther. (2009) 9:503–9. doi: 10.1586/era.09.6 19374603

[16] Jin L Liu WR Tian MX Jiang XF Wang H Zhou PY . CCL24 contributes to HCC Malignancy via RhoB- VEGFA-VEGFR2 angiogenesis pathway and indicates poor prognosis. Oncotarget. (2017) 8:5135–48. doi: 10.18632/oncotarget.14095 28042950 PMC5354897

[17] Cong Z Zhao H Zhang S You T Xie Y . LAGE3 promotes angiogenesis on hepatocellular carcinoma by stabilizing VEGFA mRNA. Biochim Biophys Acta Mol Basis Dis. (2024) 1870:167196. doi: 10.1016/j.bbadis.2024.167196 38653358

[18] Paskeh MDA Ghadyani F Hashemi M Abbaspour A Zabolian A Javanshir S . Biological impact and therapeutic perspective of targeting PI3K/Akt signaling in hepatocellular carcinoma: Promises and challenges. Pharmacol Res. (2023) 187:106553. doi: 10.1016/j.phrs.2022.106553 36400343

[19] Zheng J Wang S Xia L Sun Z Chan KM Bernards R . Hepatocellular carcinoma: Signaling pathways and therapeutic advances. Signal Transduct Target Ther. (2025) 10:35. doi: 10.1038/s41392-024-02075-w 39915447 PMC11802921

[20] Song J Guan Z Song C Li M Gao Z Zhao Y . Apatinib suppresses the migration, invasion and angiogenesis of hepatocellular carcinoma cells by blocking VEGF and PI3K/AKT signaling pathways. Mol Med Rep. (2021) 23:429. doi: 10.3892/mmr.2021.12068 33846786 PMC8047914

[21] Xie Z Wu Y Peng N Wang J Wang H Zhao L . BAIAP2L2 facilitates hepatocellular carcinoma progression and immune evasion of via targeting JAK1-mediated pathway and PD-L1 expression. Cancer Gene Ther. (2025) 32:464–74. doi: 10.1038/s41417-025-00890-z 40097840

[22] Fu X Sun G Tu S Fang K Xiong Y Tu Y . Hsa_circ_0046523 mediates an immunosuppressive tumor microenvironment by regulating MiR-148a-3p/PD-L1 axis in pancreatic cancer. Front Oncol. (2022) 12:877376. doi: 10.3389/fonc.2022.877376 35712476 PMC9192335

[23] Liu Z Tao J Zhu Y Li D Teng L . Silencing CXCR6 promotes epithelial-mesenchymal transition and invasion in colorectal cancer by activating the VEGFA/PI3K/AKT/mTOR pathway. Int Immunopharmacol. (2024) 143:113529. doi: 10.1016/j.intimp.2024.113529 39500082

[24] Wu Y Wang B Mao X Chen W Akber Aisa H . Harmine derivative B-9-3 inhibits non-small cell lung cancer via the VEGFA/PI3K/AKT pathway. Front Pharmacol. (2025) 16:1526952. doi: 10.3389/fphar.2025.1526952 40432889 PMC12107193

[25] Kou Y Zhu R Gu F Tang H Yang R Wang Y . Dioscin suppresses epithelial-mesenchymal transition in gastric cancer by upregulating Cx43 to inhibit the VEGFA/PI3K/Akt/mTOR pathway. Phytomedicine. (2026) 150:157646. doi: 10.1016/j.phymed.2025.157646 41353880

[26] Lv X Tan X Xiao Z Chen W Xu Y . TCF3 activates VEGFA transcription and reinforces PD-L1 expression in lung adenocarcinoma cells via NF-κB to attenuate the cytotoxicity of CD8(+) T cells. Expert Rev Clin Immunol. (2025) 21:1685–98. doi: 10.1080/1744666x.2025.2585347 41216989

[27] Kinsey E Lee HM . Management of hepatocellular carcinoma in 2024: The multidisciplinary paradigm in an evolving treatment landscape. Cancers (Basel). (2024) 16:666. doi: 10.3390/cancers16030666 38339417 PMC10854554

[28] Abdelhamed W El-Kassas M . Hepatocellular carcinoma recurrence: Predictors and management. Liver Res. (2023) 7:321–32. doi: 10.1016/j.livres.2023.11.004 39958776 PMC11791921

[29] Huang C Zeng Q Chen J Wen Q Jin W Dai X . TMEM160 inhibits KEAP1 to suppress ferroptosis and induce chemoresistance in gastric cancer. Cell Death Dis. (2025) 16:287. doi: 10.1038/s41419-025-07621-0 40223081 PMC11994801

[30] Morse MA Sun W Kim R He AR Abada PB Mynderse M . The role of angiogenesis in hepatocellular carcinoma. Clin Cancer Res. (2019) 25:912–20. doi: 10.1158/1078-0432.Ccr-18-1254 30274981

[31] Pinto E Pelizzaro F Farinati F Russo FP . Angiogenesis and hepatocellular carcinoma: From molecular mechanisms to systemic therapies. Med (Kaunas). (2023) 59:1115. doi: 10.3390/medicina59061115 37374319 PMC10305396

[32] Yao DF Wu XH Zhu Y Shi GS Dong ZZ Yao DB . Quantitative analysis of vascular endothelial growth factor, microvascular density and their clinicopathologic features in human hepatocellular carcinoma. Hepatobiliary Pancreat Dis Int. (2005) 4:220–6. 15908319

[33] Yang W Li Z Qin R Wang X An H Wang Y . YY1 promotes endothelial cell-dependent tumor angiogenesis in hepatocellular carcinoma by transcriptionally activating VEGFA. Front Oncol. (2019) 9:1187. doi: 10.3389/fonc.2019.01187 31799179 PMC6868052

[34] Yan JJ Zhang YN Liao JZ Ke KP Chang Y Li PY . MiR-497 suppresses angiogenesis and metastasis of hepatocellular carcinoma by inhibiting VEGFA and AEG-1. Oncotarget. (2015) 6:29527–42. doi: 10.18632/oncotarget.5012 26336827 PMC4745744

[35] Tian LY Smit DJ Jücker M . The role of PI3K/AKT/mTOR signaling in hepatocellular carcinoma metabolism. Int J Mol Sci. (2023) 24:2652. doi: 10.3390/ijms24032652 36768977 PMC9916527

[36] Sun EJ Wankell M Palamuthusingam P McFarlane C Hebbard L . Targeting the PI3K/Akt/mTOR pathway in hepatocellular carcinoma. Biomedicines. (2021) 9:1639. doi: 10.3390/biomedicines9111639 34829868 PMC8615614

[37] Cheng S Zhang X Xu Y Dai X Li J Zhang T . Krüppel-like factor 8 regulates VEGFA expression and angiogenesis in hepatocellular carcinoma. Sci Rep. (2018) 8:17415. doi: 10.1038/s41598-018-35786-6 30479372 PMC6258679

[38] Huang DF Zhang WJ Chen J Jiao ZG Wang XL Rao DY . Hepatocellular carcinoma cell-derived small extracellular vesicle-associated CD147 serves as a diagnostic marker and promotes endothelial cell angiogenesis via the PI3K/Akt pathway. Extracell Vesicles Circ Nucl Acids. (2023) 4:532–47. doi: 10.20517/evcna.2023.30 40357132 PMC12066417

[39] Lei Z Luo Y Lu J Fu Q Wang C Chen Q . FBXO22 promotes HCC angiogenesis and metastasis via RPS5/AKT/HIF-1α/VEGF-A signaling axis. Cancer Gene Ther. (2025) 32:198–213. doi: 10.1038/s41417-024-00861-w 39809956 PMC11839479

[40] Hao L Li S Deng J Li N Yu F Jiang Z . The current status and future of PD-L1 in liver cancer. Front Immunol. (2023) 14:1323581. doi: 10.3389/fimmu.2023.1323581 38155974 PMC10754529

[41] Kuang L Pang Y Fang Q . TMEM101 expression and its impact on immune cell infiltration and prognosis in hepatocellular carcinoma. Sci Rep. (2024) 14:31847. doi: 10.1038/s41598-024-83174-0 39738479 PMC11686260

[42] Sun P Xu H Guo C Yang L Zhang X Lu B . TMEM115 as an oncogenic and immunological biomarker in hepatocellular carcinoma. Liver Int. (2025) 45:e70048. doi: 10.1111/liv.70048 40052693

[43] Hill M Russo S Olivera D Malcuori M Galliussi G Segovia M . The intracellular cation channel TMEM176B as a dual immunoregulator. Front Cell Dev Biol. (2022) 10:1038429. doi: 10.3389/fcell.2022.1038429 36340035 PMC9630633

[44] Segovia M Russo S Jeldres M Mahmoud YD Perez V Duhalde M . Targeting TMEM176B enhances antitumor immunity and augments the efficacy of immune checkpoint blockers by unleashing inflammasome activation. Cancer Cell. (2019) 35:767–781.e6. doi: 10.1016/j.ccell.2019.04.003 31085177 PMC6521897

[45] Zheng Y Zhao Y Jiang J Zou B Dong L . Transmembrane protein 100 inhibits the progression of colorectal cancer by promoting the ubiquitin/proteasome degradation of HIF-1α. Front Oncol. (2022) 12:899385. doi: 10.3389/fonc.2022.899385 35928881 PMC9343598

[46] Walker TJ Reyes-Alvarez E Hyndman BD Sugiyama MG Oliveira LCB Rekab AN . Loss of tumor suppressor TMEM127 drives RET-mediated transformation through disrupted membrane dynamics. Elife. (2024) 12:RP89100. doi: 10.7554/eLife.89100 38687678 PMC11060712

[47] Chai ZT Kong J Zhu XD Zhang YY Lu L Zhou JM . MicroRNA-26a inhibits angiogenesis by down-regulating VEGFA through the PIK3C2α/Akt/HIF-1α pathway in hepatocellular carcinoma. PloS One. (2013) 8:e77957. doi: 10.1371/journal.pone.0077957 24194905 PMC3806796

